# Repeated exposure decreases aesthetic chills likelihood but increases intensity

**DOI:** 10.1371/journal.pone.0300494

**Published:** 2025-04-02

**Authors:** Felix Alexandre Schoeller, Leonardo Christov-Moore, Caitlin Lynch, Abhinandan Jain, Thomas Diot, Nicco Reggente

**Affiliations:** 1 Institute for Advanced Consciousness Studies, Santa Monica, California, United States of America; 2 MIT Media Lab, Cambridge, Massachusetts, United States of America; 3 Department of Psychiatry, GHU Paris Psychiatrie et Neurosciences, Paris, France; University of Pecs Medical School, HUNGARY

## Abstract

Aesthetic chills are a peak emotional response to stimuli such as music, films, or speech characterized by shivers and goosebumps and activation of dopaminergic circuits. Despite growing scientific interest in this phenomenon, repeated exposure to chills stimuli has not been studied yet, due to the absence of a validated database. This study leverages a recent gold standard in chills stimuli to investigate the impact of repeated exposure on the frequency and intensity of aesthetic chills. Participants (n = 58) were randomly exposed to 6 chill-evoking stimuli pre-validated on the population of interest, in a counterbalanced order. Our findings revealed a significant decrease in the likelihood of experiencing chills with repeated exposure, suggesting habituation to chills itself or potential fatigue in response to aesthetic stimuli. However, we observed an increase in the intensity and duration of chills over successive exposures among those who did experience chills. The study also identified distinct demographic and psychophysiological response patterns across different participant groups, indicating variability in chill responses. These results provide insights into the dynamic nature of aesthetic experiences and their underlying neural mechanisms, with implications for understanding emotional and reward processing in psychophysiology.

## 1. Introduction

Aesthetic chills (henceforth “chills”) are an intense pleasurable experience characterized by distinct physiological markers including piloerection and changes in skin conductance, heart rate, respiration, and body temperature [1–5]. Chills typically occur during peak emotional responses to stimuli like music [6], films [7], and speeches [8], engaging brain regions linked to reward/avoidance and dopamine release [6,9]. Recent evidence suggests chills may have therapeutic potential for disorders involving dopamine dysfunction such as depression [10–13]. However, existing chills research relies heavily on self-selected musical excerpts, limiting generalizability and introducing pre-selection biases [14]. Crucially, the longitudinal dynamics of chills responses remain poorly characterized, hindering translation into personalized clinical applications. Peak emotional states like chills are susceptible to dynamic changes over time, where repeated exposure may attenuate or amplify the intensity of the response over time [15]. Quantifying such effects constitutes a key step in developing interventions leveraging short-term alterations in consciousness for long-term clinical benefits.

In this first preliminary study, we assessed changes in chills experiences during repeated exposure to chills-evoking audiovisual stimuli. We leveraged a set of six pre-validated chill-evoking audiovisual stimuli, sourced from ecologically valid platforms (YouTube, Reddit) and empirically validated prior across 3,500 + listeners [16]. A focus was placed on music and speech given their engagement of reward and emotional circuits [17,18]. To explore the consequences of repeated chills exposure, we quantified longitudinal changes in chills likelihood and parameters (frequency, intensity, duration). Note that classical habituation paradigms typically use the same stimulus repeatedly [19], here in contrast we employed different stimuli across exposure trials. While all stimuli were pre-validated to evoke chills, they represented distinct audiovisual experiences. Any observed changes may not strictly constitute habituation, but could emerge from generalized fatigue, sensory adaptation, or other processes when exposed to varied stimuli that engage overlapping neural circuits. We hypothesized that chills likelihood and intensity would decrease with repeated exposure due to habituation. Characterizing such dynamics constitutes a critical first step towards developing positive affect exposure interventions for disorders linked to blunted reward sensitivity.

## 2. Methods

### 2.1. Participants

58 participants from Southern California were recruited online using the Prolific platform [20]. Most participants identified as White or Caucasian (63.79%, N = 37) and had a Bachelor’s degree (50.0%, N = 29). A common income level was “$75,000-$99,999” (22.41%, N = 13). There was a fairly even mix of Male (50.0%, N = 29) and Female (44.83%, N = 26) participants, with a small number identifying as Non-Binary/Fluid (5.17%, N = 3). Participants were recruited through an online platform with comprehensive pre-screening features commonly used to recruit participants (Prolific). Prolific is a specialized online platform designed to connect researchers with a global pool of participants for research studies, offering tailored participant recruitment through a range of pre-screening tools including mental health diagnoses, medication, age, gender identity, nationality, and employment status [20]. Participants were English-speaking, California residents with U.S. nationality with no hearing difficulties or history of neurologic disorder. All participants underwent the whole procedure, no participant was excluded from the analysis. Participants were compensated $12 per hour.

### 2.2. Stimuli

The stimuli utilized in this study were sourced from ChillsDB, a comprehensive database of chill-inducing stimuli that have been empirically validated across a sample of over 3,000 participants from Southern California [16]. Stimuli spanning various modalities (films, music, speech) were selected to ensure a wide-ranging exploration of chills, while also considering the varied chills ratio associated with each stimulus (Table 1).

**Table 1:** The table provides details about the six different videos used in the study. For each video, a brief description, its length, and its chills ratio (CR) from past ChillsDB studies are included. The CR tells us how often chills were experienced by viewers in past studies. The descriptions give a quick idea about what each video is about and what viewers might experience.

Stimulus	Description (including duration, chills ratio)
Hallelujah (6:26, CR = .7)	Choir! Choir! Choir! began as a weekly drop-in singing event, equal parts singing, comedy, and community-building. In 2015, 1500 singers came to Luminato Festival in Toronto for a very special event. Choir! leaders taught them backup parts to Leonard Cohen’s Hallelujah, and Rufus Wainwright joined the crowd on stage to lead the vocals. The collective performance amplifies the power of the musical piece.
Great Dictator (4:11, CR = .58)	In the 1940 satire-drama The Great Dictator, Charlie Chaplin plays 2 doppelganger characters: a fascist dictator and a Jewish barber avoiding persecution. In this scene, the Barber is mistaken for the Dictator and must give a speech. Revered as one of the greatest monologues in film history, Chaplin makes a call for brotherhood and goodwill, encouraging soldiers to fight for liberty, and unite people in the name of democracy. What you are about to hear is the voice of Charlie Chaplin from the middle of World War II. For his entire career, he had been a silent actor. But in 1940, he decided to speak out. This is his message of hope.
Unbroken (5:57, CR = .56)	This motivational compilation, from a series by Youtube creator Mateusz M., combines quotes from Les Brown, Steve Jobs, Eric Thomas, and Louis Zamperini, with scenes from Her and Jobs to make a single motivational message. It is intended to encourage the listener to take full responsibility for one’s life and follow one’s path, even when it’s unconventional; to pursue what one loves, and live a principled life in the face of hardship.
We think too much and feel too little (5:27, CR = .53)	This inspirational content, from Youtube creator Slyfer2812, combines scenes from a number of films (including Birdman, Chaplin, I am a legend, Into the Wild, Moonlight, The Greatest Showman, The Theory Of Everything, and Iron Man 3) to create a single motivational message. It is intended to encourage the audience to reflect on the importance of relationships and humans’ profound connection to everything, inspiring not just to survive but to truly live.
Agnus Dei (7:22 = .51)	The Flemish Radio Choir performs Samuel Barber’s Agnes Dei, Opus 11, in Brussels. Agnus Dei (“Lamb of God”) comes from John 1:29 and is a part of the Latin liturgical tradition. The words of the song are a request for peace and forgiveness. In 1967, Barber set the Latin words of the liturgical Agnus Dei, a part of the Mass, for mixed chorus with optional organ or piano accompaniment.
Silva (2:37, CR = .5)	Storyteller Jason Silva considers the impermanence of all and the dual nature of human consciousness, through a meditation on a passage from Ernest Becker: “Man is literally split in two: he has an awareness of his own splendid uniqueness in that he sticks out of nature with a towering majesty, and yet he goes back into the ground a few feet in order blindly and dumbly to rot and disappear forever” -Ernest Becker

### 2.3. Procedure

Participants were recruited via the Prolific platform, targeting individuals in Southern California. Upon recruitment, participants were redirected to a webpage specifically designed to host the experiment. The experiment began with the collection of demographic data, where participants responded to questions concerning their race, education level, household income, age, and sex, aligning with established demographic query standards. Following demographic data collection, participants were prompted to report their initial emotional and psychological state by responding to questions concerning their current valence, arousal, tension, and energy levels. Participants were then randomly exposed to one of the six chills stimuli. After engaging with the stimulus, participants were requested to answer questions pertaining to their chills experience, explicitly probing into the frequency, intensity, and duration of the chills experienced during the stimulus exposure. Additional questions concerning physiological responses (such as tears and goosebumps), as well as their overall enjoyment and liking of the video, were also posed to the participants. The intertrial interval between successive stimuli exposures lasted approximately 3 minutes to allow participants to complete the post-stimulus questionnaires. This cycle of stimulus exposure followed by post-stimulus questioning was repeated for all six stimuli. The order of stimuli presentation was randomized to control for potential order effects and to ensure the generalizability of the findings. After the final stimulus exposure and corresponding questions, participants were prompted to report their emotional and psychological state, capturing their valence, arousal, tension, and energy levels post-experiment. Concluding the experiment, participants responded to open-ended questions concerning their perspectives on the length and complexity of the questionnaire. Additionally, participants were provided with contact information through which to learn more about the chills phenomenon and the aims and hypotheses of the study. Participant feedback was predominantly positive, indicating good reception and engagement with the experiment. The entire experimental session lasted an average of 48 minutes.

### 2.4. Questionnaires

We employed a 10-item scale used to assess participants’ emotional valence, arousal, tension, and energy, following the positive and negative activation (PA/NA) framework pioneered by Tellegen et al. [21]. This scale evaluates positive and negative activation, along with valence (emotional pleasure), and is commonly used in experience sampling method (ESM) studies for quick, reliable state assessments in everyday situations (“How excited/pleasant/tense/energetic do you feel at the moment?”).

Chills were self-reported by the participants through a series of questions regarding their emotional and physiological responses to the stimulus. They responded to binary (yes/no) questions such as “Did you experience chills?” and “Did you experience goosebumps?”, as well as questions about the frequency and intensity of chills rated on a 0-10 Likert scale. Additionally, a qualitative component involved open-ended responses, asking participants to describe their experience during the video, their description of what caused the chills in the video, and whether the video reminded them of a personal experience, providing a deeper insight into their emotional engagement with the content. Additional variables collected included the presence of tears (yes/no), goosebumps (yes/no), and liking/enjoyment of each stimulus (0-10 scale). However, these variables were not analyzed for the present study.

### 2.5. Statistical analyses

Linear regression models were constructed to examine the relationships between presentation order and chills intensity, frequency, and duration. Models included presentation order as the predictor and the various chills parameters as outcomes. Standard regression diagnostics were performed, including analyses of residuals.

A multivariate logistic regression model was created to analyze the effects of presentation order, valence, and arousal on the likelihood of chills, with individual participant and specific stimuli coded as random effects. Model fit was assessed using Akaike’s Information Criterion (AIC). To categorize participants based on psychological response patterns, a hierarchical cluster analysis was conducted on trajectories of valence, arousal, tension, and energy ratings across stimulus presentations. Ward’s linkage method with squared Euclidean distances was used to generate clusters. Differences between clusters were analyzed via ANOVA and chi-squared tests. For all analyses, statistical significance was defined as p <.05. Estimates are reported alongside standard errors and test statistics. Effect sizes are indicated using R-squared values for regression analyses and odds ratios for the logistic regression. Plots visualizing key relationships supplement the analyses. The analyses were conducted using the R software (version 4.3.2).

### 2.5. Ethics

The experiment is in compliance with the Helsinki Declaration. Following review, the study protocol was granted an exemption status by Advarra IRB (Pro00068209). All participants gave their voluntary informed consent and we followed the Ethics Code of the American Psychological Association. All participants were informed about the purpose of the research, their right to decline to participate and to withdraw from the experiment, and the limits of confidentiality. We also provided them with a contact for any questions concerning the research and with the opportunity to ask any questions regarding the phenomenon under study (aesthetic chills) and receive appropriate answers.

## 3. Results

### 3.1. Chills likelihood decreases with presentation order

**Fig 1:**
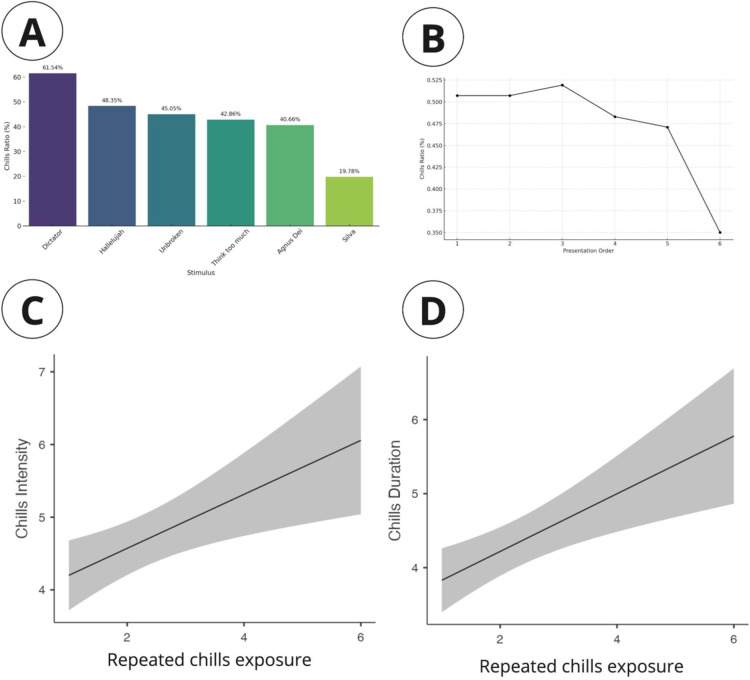
A. Bar chart showing the percentage of participants experiencing chills in response to various stimuli, ordered by descending frequency. B: Line graph indicating a decrease in the chills ratio over successive presentations of stimuli, with a marked drop between the fifth and sixth exposure. C: Plot illustrating the positive correlation between the intensity of chills and the number of repeated exposures to chills stimuli. D: Graph depicting an increase in the duration of chills with repeated exposure to the stimuli.

After creating several univariate models for each of the four variables (valence, arousal, tension, and energy), we constructed a multivariate model using the Akaike Information Criterion (AIC) as a statistical criterion. This model incorporates the stimulus presentation order, valence, and arousal as significant factors. The likelihood of experiencing chills decreased with the number of stimuli presented (Fig 1). Conversely, it increased when individuals exhibited higher levels of valence and arousal (p < 0.01). This result takes into account the difference between individuals and the various stimuli received by each.

We conducted a linear regression analysis examining the relationship between presentation order and the intensity of chills among participants who experienced chills; a significant effect was found. The estimate for presentation order was 0.371 with a standard error of 0.129, resulting in a t-value of 2.89 and a p-value of 0.004. The intercept was also significant with an estimate of 3.829, a standard error of 0.343, and a t-value of 11.18 (p <.001). The model explained approximately 4.5% of the variance in chills intensity (R-squared = 0.0450), indicating a modest overall fit with a correlation coefficient of 0.212.

For chills frequency, the linear regression analysis indicated that presentation order did not significantly predict frequency (estimate = 0.0653, standard error = 0.0509, t = 1.28, p = 0.201). The intercept was significant (estimate = 1.8586, standard error = 0.1353, t = 13.73, p <.001). The model’s R-squared was 0.00918, suggesting that only about 0.918% of the variance in chills frequency was accounted for by the presentation order.

Regarding chills duration, the analysis showed a significant effect of presentation order (estimate = 0.390, standard error = 0.115, t = 3.37, p <.001). The intercept was significant as well (estimate = 3.440, standard error = 0.307, t = 11.19, p <.001). The model explained about 6.01% of the variance in chills duration (R-squared = 0.0601), with a correlation coefficient of 0.245 (Table 2).

**Table 2:** Multivariable mixed effect logistic regression for chills, with individual ID and stimulus as a random effect. Individual intercept variance = 1.02 and stimuli intercept variance = 0.62 (Number of observations: 346. ID groups: 58; Stimulus groups: 6).

	OR [95%CI] for chills	p-value
Intercept	0.22 [0.06; 0.79]	**0.02**
Stimulus order	0.77 [0.66; 0.9]	** < 0.01**
Valence	1.26 [1.09; 1.46]	** < 0.01**
Arousal	1.24 [1.09; 1.4]	** < 0.01**

### 3.2. Three distinct groups in chill response

**Fig 2:**
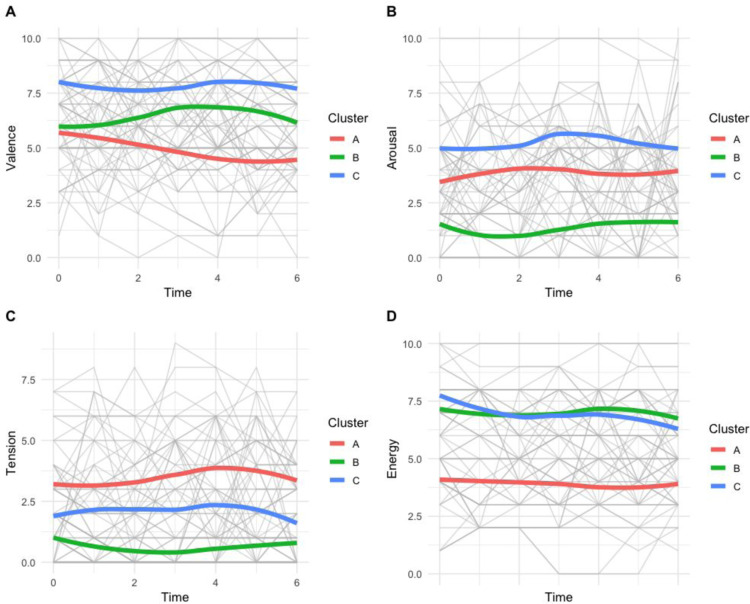
Evolution of the four pre-stimuli psychological parameters (A: Valence, B: Arousal, C: Tension, D: Energy). Each color represents one cluster, and the grey line represents individual evolution.

The analysis of trajectories of psychological parameters before and after each stimulus identified three distinct groups (Fig 2, Table 3). The first group (cluster A) is characterized by an average level of chills, with the lowest levels of valence and energy, and the highest level of tension. This group is primarily composed of women (p < 0.01) with a high level of education. Additionally, this group stands out for having the highest average duration of chills before any stimulation. The second group (cluster B) exhibits the lowest rate of chills, with low levels of tension and arousal and a high level of energy. This group consists mostly of men with a low level of education. They also had the shortest average duration of chills before stimulation. The last group (cluster C) displays the highest rate of chills during the experience and is characterized by very high levels of valence, arousal, and energy. This group comprises older white men (average age 45) with a relatively high level of education.

**Table 3:** Comparative Analysis of Chills Experience Across Three Groups: This table presents a detailed comparison of three groups (A, B, and C) in terms of their experiences with chills. It includes the number of participants in each group, frequency, intensity, and duration of chills, along with demographics such as gender, age, race, education level, and income. Statistical significance (p-value) is also provided for each category.

	A (n = 29)	B (n = 16)	C (n = 13)	p
**Number of chills**				**0.02**
Mean (SD)	2.5 (1.5)	2.1 (1.5)	3.8 (1.8)	
Median [Q1, Q3]	2.0 [2.0, 3.0]	2.0 [1.5, 3.2]	4.0 [3.0, 5.0]	
**Gender**				**0.01**
Female	18 (62.1%)	4 (25.0%)	4 (30.8%)	
Male	8 (27.6%)	12 (75.0%)	9 (69.2%)	
Non-Binary/Fluid	3 (10.3%)	0 (0.0%)	0 (0.0%)	
**Age**	33.8 (12.7)	34.0 (11.1)	44.5 (14.6)	**0.04**
**Races that you consider yourself to be**				0.49
Black or African American, Asian, Other	5 (17.2%)	6 (37.5%)	2 (15.4%)	
White + Other	5 (17.2%)	1 (6.2%)	2 (15.4%)	
White or Caucasian	19 (65.5%)	9 (56.2%)	9 (69.2%)	
**Highest level of education**				**0.02**
Associates or technical degree	1 (3.4%)	2 (12.5%)	4 (30.8%)	
Bachelor’s degree, graduate or professional degree (MA, MS, MBA, PhD, JD, MD, DDS etc.)	19 (65.5%)	7 (43.8%)	6 (46.2%)	
High school diploma or GED	7 (24.1%)	1 (6.2%)	2 (15.4%)	
High school or some college, but no degree	2 (6.9%)	6 (37.5%)	1 (7.7%)	
**Total household income before taxes during the past 12 months**				0.96
Less than $25,000	5 (17.9%)	3 (18.8%)	2 (15.4%)	
$25,000-$49,999	4 (14.3%)	4 (25.0%)	2 (15.4%)	
$50,000-$74,999	8 (28.6%)	1 (6.2%)	3 (23.1%)	
$75,000-$99,999	6 (21.4%)	4 (25.0%)	3 (23.1%)	
$100,000-$149,999	3 (10.7%)	2 (12.5%)	2 (15.4%)	
$150,000 or more	2 (7.1%)	2 (12.5%)	1 (7.7%)	
**Chills frequency**				0.20
More than Once a week	14 (48.3%)	3 (18.8%)	3 (23.1%)	
Never or less than Once a year	4 (13.8%)	3 (18.8%)	4 (30.8%)	
Once a month	11 (37.9%)	10 (62.5%)	6 (46.2%)	
**Chills intensity**	5.8 (1.5)	5.0 (1.9)	5.3 (2.5)	0.40
**Chills duration**	3.7 (1.3)	2.6 (1.0)	3.4 (1.6)	**0.03**
**Chills like**	5.4 (2.5)	5.6 (2.6)	5.1 (2.8)	0.88

## 4. Discussion

We found a decline in chills likelihood with repeated stimulus exposure. Specifically, a decreasing trend in chills likelihood across stimulus presentations, with a notable drop from the fifth to the sixth stimulus. This suggests potential adaptation of the neurophysiological mechanisms supporting chills, consistent with existing evidence on the effects of repeated exposure on other reward-related responses [22]. By this interpretation, the observed decline likely reflects dynamic adjustments in neural response over repeated exposures, reducing sensitivity of reward pathways. While this study does not directly test the mechanisms underlying these effects, potential neural adaptation processes could include changes in receptor activity or postsynaptic signaling cascades [23]. Importantly, we acknowledge that the study design does not align with a traditional habituation paradigm, as it involves varied stimuli rather than repeated exposure to the same stimulus. Future research could investigate whether the decline in response represents habituation by employing a design that repeatedly exposes participants to identical stimuli within or across sessions, following the principles of habituation paradigms [19]. This approach would provide clearer insights into whether the observed effects stem from habituation or broader adaptation processes.

These results also underscore the relationship between chills and pre-stimulus psychological states [24]. We found that higher valence and arousal levels correlate with increased chills likelihood. This aligns with prior evidence linking chills to physiological arousal and positive emotions [2,4]. Our analysis of psychological trajectories revealed groups exhibiting positive arousal changes post-listening also had higher chills frequencies. This further supports chills’ role as a marker of strong emotive responses to aesthetic stimuli. Notably, while the overall sample showed habituation effects, a subset of participants experienced consistent chills with heightened intensity across repetitive exposures. This points to meaningful individual differences in reward pathway excitability and aesthetically-driven emotional reactivity. Integrating neural and phenotypic data could elucidate if the sensitivity stems from endogenous factors like genotype or from external sociocultural exposure.

While the overall likelihood of experiencing chills decreased with successive presentations, both the intensity and duration of chills actually increased among those who continued to experience them. The declining trend in chills likelihood, with a notable drop from the fifth to sixth stimulus, aligns with the concept of habituation, where repeated exposure leads to a gradual decrease in response strength [15]. However, the concurrent increase in chills intensity and duration among responders deviates from typical habituation patterns and suggests a sensitization effect [24]. This divergence between response likelihood and magnitude parameters is a novel finding that may be specific to appetitive, reward-related responses like aesthetic chills.

One interpretation is that repeated exposure to chills-eliciting stimuli leads to a general habituation of the chills response trigger, reducing how often it is activated. However, in cases where the response threshold is still surpassed, the prior stimulation primes or sensitizes the neural circuits, resulting in a heightened response intensity and duration. This dual process could reflect an adaptive mechanism to prevent overstimulation while still maintaining responsiveness to particularly salient or powerful stimuli. Elucidating the rate of decay of the chills response and its neural correlates can provide key insights into these processes and their implications for reward learning and aesthetic emotion.

Notable limitations should be considered. First, our investigation focused solely on audiovisual stimuli that induce aesthetic chills responses. While this allowed us to examine the specific dynamics of chills to this type of multisensory stimuli, the results may not generalize to other modalities that can elicit chills, such as purely auditory, visual, or even tactile stimuli without multisensory integration [8,13,25]. Additionally, while we did find distinct patterns across demographic groups in our sample, the participants were recruited from a geographically limited region (Southern California). This relatively homogeneous sample in terms of cultural background may limit the generalizability of our findings to more diverse populations. Future studies should explore potential cultural influences [1,26]. While our analysis revealed distinct subgroups based on psychological response patterns, the limited sample size within each subgroup precluded a reliable examination of how the atypical habituation effects (decreased chills likelihood but increased intensity and duration) may have manifested differentially across these subgroups. Future studies with larger samples should investigate potential subgroup differences in the dynamics of chills responses over repeated exposures. Finally, our sample reflected a higher education and income level compared to the general population, so replication in a more socioeconomically diverse sample is warranted.

## 5. Conclusion

This study investigated the impact of repeated exposure to emotionally charged stimuli on the likelihood, intensity, and duration of aesthetic chills. The results revealed a significant decrease in the likelihood of experiencing chills with successive presentations of the stimuli. However, among participants who continued to experience chills, there was an increase in the intensity and duration of the response over repeated exposures. These findings provide insights into the dynamic nature of aesthetic responses and the complex interplay of habituation and sensitization processes that may shape them over time. Future research should aim to replicate and extend these findings across diverse populations, stimulus types, and modalities while integrating neuroimaging and physiological measures to elucidate the underlying mechanisms.
